# A clinical algorithm to identify people with the glucose-6-phosphate dehydrogenase p.Val68Met variant at risk for diabetes undertreatment

**DOI:** 10.1016/j.gimo.2025.103468

**Published:** 2025-10-24

**Authors:** Yash Pershad, Joseph H. Breeyear, Robert W. Corty, Jonathan D. Mosley, VA Million Veteran Program, Lawrence S. Phillips, Ayush Giri, Dan M. Roden, Todd L. Edwards, Alexander G. Bick

**Affiliations:** 1 Department of Medicine, Vanderbilt University Medical Center, Nashville, TN; 2 Vanderbilt Genetics Institute, Vanderbilt University, Nashville, TN; 3 Biostatistics and Computational Biology Branch, Division of Intramural Research, National Institute of Environmental Health Sciences, Durham, NC; 4 Department of Medicine, University of Texas Southwestern Medical Center, Dallas, TX; 5 Atlanta VA Health Care System, Decatur, GA; 6 Division of Endocrinology and Metabolism, Department of Medicine, Emory University School of Medicine, Atlanta, GA; 7 VA Tennessee Valley Healthcare System, Nashville, TN; 8 Department of Biomedical Informatics, Vanderbilt University Medical Center, Nashville, TN

**Keywords:** Clinical algorithm, Genomic learning health system, Glucose-6-phosphate dehydrogenase deficiency, Hemoglobin-A1c, Personalized medicine

## Abstract

**Purpose:**

To develop an algorithm using routine clinical laboratory measurements to identify people at risk for systematic underestimation of glycated hemoglobin because of p.Val68Met glucose-6-phosphate dehydrogenase (G6PD) deficiency.

**Methods:**

We analyzed 122,307 participants of self-identified Black race across 4 large cohorts with blood glucose, glycated hemoglobin, and red cell distribution width measurements from a single blood draw. In UK Biobank, we used recursive partitioning to train 2 models to predict possible and likely G6PD deficiency. We validated the algorithm in NIH AllofUs, Vanderbilt BioVU, and the Million Veterans Program. In the Vanderbilt Synthetic Derivative with clinical but no genetic data, we created a cohort of 48,031 participants with type 2 diabetes and no genetic data to test whether predicted risk for G6PD deficiency was associated with incident diabetic retinopathy.

**Results:**

G6PD deficiency predictions in hemizygous males showed precision/recall of 31%/81% for possible and 81%/10% for likely deficiency. In homozygous females, precision/recall was 6%/76% for possible and 34%/13% for likely deficiency. Patients with diabetes having predicted possible deficiency demonstrated 1.4-fold higher 20-year retinopathy rates (14.3% vs 11.2%, *P* = .003).

**Conclusion:**

We report a simple clinical algorithm that enables health care systems to identify people who may benefit from G6PD genotyping and glucose-based diabetes monitoring.

## Graphical abstract

**Figure:**
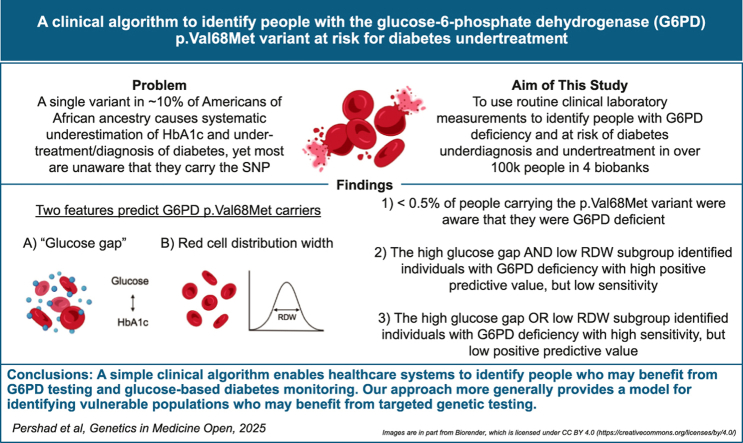

## Introduction

The promise of translating genetic insights into clinical medicine remains partially unfulfilled, particularly for historically underserved populations. The goal of a genomic learning health system (gLHS) is to identify and advance approaches for integrating genomic information into existing learning health systems. A major barrier to achieving this goal is the present inability to perform genetic testing in all people. Therefore, a gLHS requires tools to quantify the burden of undiagnosed genetic conditions within its patient populations and prioritize those who may benefit from genetic testing.

An example of this situation is glucose-6-phosphate dehydrogenase (G6PD) deficiency. This X-linked enzymopathy, which shortens erythrocyte lifespan,^1^^,^^2^ causes systematically lower glycated hemoglobin (HbA1c) relative to blood glucose,^3^^,^^4^ creating a population-level risk of diabetes underdiagnosis and undertreatment that disproportionately affects people of African ancestry.^5^ The American Diabetes Association standard of care in diabetes identifies G6PD deficiency as a major cause of interference with HbA1c reference ranges and recommends to use plasma glucose or fructosamine to diagnose and manage diabetes.^6^

Although G6PD deficiency may be diagnosed via spectrophotometry to assess G6PD enzyme activity, fluorescent spot testing to detect nicotinamide adenine dinucleotide phosphate (NADPH) produced by G6PD, or genotyping of common variants in certain populations, such as p.Val68Met in individuals of African ancestry, people with G6PD deficiency typically remain unidentified unless they experience rare hemolytic crises.^2^^,^^7^ The impact of G6PD deficiency on diabetes management and its underdiagnosis creates a critical need for a tool to identify people with high risk who should be tested.

Here, we developed an algorithm using routine laboratory measurements to estimate G6PD p.Val68Met variant prevalence and prioritize a group who would likely benefit from genotyping for the variant. The G6PD p.Val68Met variant, found almost exclusively in those of African ancestry, demonstrates how algorithms built with clinical data can help gLHSs identify vulnerable populations to prioritize initial genetic-based interventions.

## Materials and Methods

We analyzed data from 122,307 participants of self-identified Black race with concurrent blood glucose, HbA1c, and red cell distribution width (RDW) measurements across 4 cohorts: UK Biobank (*N* = 3,864), NIH AllofUs (*N* = 2,047), Vanderbilt BioVU (*N* = 2,087), and the Million Veterans Program (MVP) (*N* = 114,309). We limited our analysis within the cohorts to those of self-identified Black race because clinicians applying this algorithm will be presently unaware of genetic ancestry. Furthermore, the G6PD p.Val68Met variant is almost exclusively found in those of self-identified Black race. Zygosity of the G6PD p.Val68Met variant was determined by extracting genotype for variant rs1050828-T. Genotype was used over enzymatic testing because it was available for all participants and is not susceptible to false negatives during reticulocytosis or hemolysis.

In the UK Biobank, blood glucose (Data-Field 30740), HbA1c (Data-Field 30750), and RDW (Data-Field 30070) were measured for all participants at baseline with the same blood draw. UK Biobank participants were asked to fast before blood glucose measurements. In AllofUs, Vanderbilt BioVU, and MVP, blood glucose (OMOP ID: 3004501), HbA1c (OMOP ID: 3004410), and RDW (OMOP ID: 3019897) were obtained as part of clinical care and linked to participant data. Because these measurements were not assured to be fasting, blood draws with blood glucose measurements less than 70 mg/dL and greater than 200 mg/dL were excluded. If an AllofUs, Vanderbilt BioVU, or MVP participant had more than 1 eligible blood draw, the HbA1c, RDW, and blood glucose values from the draw with the lowest blood glucose (most likely to be fasting) was used. We calculated the glucose gap (GG) as measured blood glucose minus the estimated average glucose level as calculated from HbA1c (28.7 × HbA1c − 46.7).^8^ Clinical diagnosis of G6PD deficiency and diabetes mellitus were defined by International Classification of Diseases (ICD) codes (Supplemental Table 1).

We evaluated the algorithm’s ability to discern 3 patient populations: (1) male G6PD p.Val68Met hemizygotes, (2) female G6PD p.Val68Met homozygotes, and (3) females with at least 1 G6PD p.Val68Met allele (heterozygotes and homozygotes). Using recursive partitioning in the UK Biobank (scikit-learn v1.5.1), we trained models for “likely” G6PD deficiency (both RDW ≤ 12 fL and GG ≥ −15 mg/dL) and “possible” G6PD deficiency (either RDW ≤ 12 fL or GG ≥ −15 mg/dL). The “possible” criteria were designed to maximize sensitivity for population-level screening, accepting lower precision to minimize missed cases of G6PD deficiency.

The UK Biobank was the training cohort. We evaluated and validated the model in AllofUs, Vanderbilt BioVU, and MVP. We assessed precision ($Precision=\frac{True\;Positives}{Test\;Positives}$) and recall ($Recall=\frac{Test\;Positives}{Genotype\;Positives}$) of possible and likely G6PD deficiency criteria for each of the 3 tasks in all cohorts.^9^ For precision, true positives were defined as participants meeting the likely or possible criteria with the relevant genotype, whereas test positives were all participants meeting the likely or possible criteria regardless of genotype. For recall, true positives were defined as above, whereas genotype positives were all participants with the relevant genotype. For comparison with previously published approaches, we also calculated the fasting glucose to HbA1c ratio as described by Chang et al.^10^

We then demonstrated population-level application of the algorithm by analyzing 48,031 people of self-identified Black race with type 2 diabetes in the Vanderbilt Synthetic Derivative, which did not have genetic data available. This health system implementation allowed us to estimate the burden of potentially undiagnosed G6PD deficiency and identify subpopulations at elevated risk for retinopathy. We calculated population-level glucose gaps and applied our thresholds to estimate G6PD p.Val68Met status. Using Cox proportional hazards regression, we examined time (years) from diabetes diagnosis to incident diabetic retinopathy diagnosis, stratified by predicted G6PD deficiency burden, adjusting for age, sex, smoking (ever or never), duration of diabetes, LDL cholesterol, and hypertension diagnosis. Follow-up was censored at time of diabetic retinopathy diagnosis (ICD codes in Supplemental Table 1), death, or end of follow-up, whichever came first.

## Results

Among UK Biobank participants who self-identify as Black race, 15.4% of males were p.Val68Met hemizygotes, whereas 26.4% and 2.4% of females were heterozygotes and homozygotes, respectively. Similar frequencies were observed in AllofUs (8.8% hemizygotes, 20.6% heterozygotes, and 0.8% homozygotes), Vanderbilt BioVU (10.3% hemizygotes, 20.1% heterozygotes, and 1.8% homozygotes), and MVP (11.7% hemizygotes, 21.0% heterozygotes, and 1.4% homozygotes). Prevalence of G6PD p.Val68Met alleles was highest in the UK Biobank. Notably, <0.5% of G6PD p.Val68Met hemizygotes and homozygotes across all cohorts had a clinical G6PD deficiency diagnosis (Table 1).

**Table 1:** Laboratory characteristics, G6PD p.Val68Met variant frequencies, and clinical diagnoses across 4 cohorts, stratified by sex

Features	Training Cohort		Evaluation Cohorts					
	UK Biobank (*N* = 3864)		All of Us (*N* = 2047)		Vanderbilt BioVU (*N* = 2087)		MVP (*N* = 114,309)	
	Males (*N* = 1914)	Females (*N* = 1950)	Males (*N* = 568)	Females (*N* = 1479)	Males (*N* = 856)	Females (*N* = 1231)	Males (*N* = 99,312)	Females (*N* = 14,997)
Labs								
Hemoglobin A1c (%, median ± IQR)	5.6 ± 0.6	5.6 ± 0.6	5.9 ± 1.0	5.8 ± 0.9	5.9 ± 1.3	6.0 ± 1.4	6.0 ± 1.1	5.5 ± 0.8
Blood glucose (mg/dL, median ± IQR)	88.0 ± 14.7	86.7 ± 13.1	102.0 ± 27.0	97.0 ± 24.0	101.0 ± 28.0	101.0 ± 31.0	105.3 ± 26.7	96.8 ± 18.7
Glucose gap (mg/dL, median ± IQR)	−26.0 ± 20.1	−25.8 ± 19.5	−20.8 ± 26.9	−21.0 ± 22.8	−19.9 ± 31.8	−23.8 ± 27.3	−21.2 ± 20.6	−22.5 ± 16.6
Red cell distribution width (fL, median ± IQR)	13.8 ± 1.2	14.0 ± 1.5	13.8 ± 1.5	14.1 ± 1.9	14.0 ± 1.7	14.3 ± 2.0	14.0 ± 1.6	14.0 ± 1.5
G6PD p.Val68Met genotype								
Heterozygotes, (%)	N/A	514 (26.4%)	N/A	304 (20.6%)	N/A	248 (20.1%)	N/A	3,142 (21.0%)
Hemi/homozygotes, (%)	294 (15.4%)	46 (2.4%)	50 (8.8%)	12 (0.8%)	88 (10.3%)	22 (1.8%)	11,610 (11.7%)	208 (1.4%)
Clinical Diagnoses								
G6PD Deficiency, (%)	0 (0.0%)	0 (0.0%)	0 (0.0%)	5 (0.3%)	3 (0.4%)	1 (0.1%)	N/A	N/A
Diabetes Mellitus, (%)	106 (5.5%)	72 (3.7%)	314 (55.3%)	738 (49.9%)	484 (56.5%)	771 (62.6%)	32,802 (33.0%)	3391 (22.6%)

Values are presented as median ± interquartile range (IQR) for laboratory measurements and *N* (%) for genotypes and diagnoses. The glucose gap represents the difference between measured blood glucose and HbA1c-estimated glucose. N/A indicates not applicable for male heterozygote status because *G6PD* is on chromosome X and the clinical diagnoses of G6PD deficiency in MVP were not available.

In the UK Biobank, RDW and GG showed strong discriminative ability for male hemizygotes (area under the receiver-operator curve (AUROC): 0.82 ± 0.03) and female homozygotes (AUROC: 0.78 ± 0.04), with moderate performance for either female heterozygotes or homozygotes (AUROC: 0.63 ± 0.02). Using recursive partitioning in the UK Biobank, we identified optimal thresholds of RDW ≤ 12 fL and GG ≥ −15 mg/dL for detecting G6PD deficiency.

We defined 2 classification thresholds: “possible” G6PD deficiency (meeting either criterion) or “likely” G6PD deficiency (meeting both criteria). For male hemizygotes, possible G6PD deficiency showed high recall (81.0%) with moderate precision (31.1%), whereas likely G6PD deficiency offered higher precision (80.8%) but lower recall (9.9%). Among females, possible G6PD deficiency identified heterozygotes or homozygotes with 50.3% recall and 43.3% precision, whereas likely G6PD deficiency showed 3.1% recall and 65.6% precision. For female homozygotes, recall and precision for possible G6PD deficiency were 76.2% and 6.2% and for likely G6PD deficiency were 13.4% and 33.9% (Table 2).

**Table 2:** Precision and recall by sex, zygosity, and cohort for (1) possible G6PD deficiency (G6PDdef) defined as red cell distribution width ≤ 12 fL or glucose gap ≥ −15 mg/dL, and (2) likely G6PD deficiency (G6PDdef) defined as red cell distribution width ≤ 12 fL and glucose gap ≥ −15 mg/dL

Sex	G6PD p.Val68Met Zygosity	Cohort	Prevalence (%)	Possible G6PD Deficiency		Likely G6PD Deficiency	
				Recall (%)	Precision (%)	Recall (%)	Precision (%)
Male	Hemizygote	Overall	11.7	81.0	31.1	9.9	80.8
		UKB	15.4	77.9	49.2	3.4	71.4
		AoU	8.8	84.0	18.6	8.0	44.4
		BioVU	10.3	80.7	20.2	11.4	66.7
		MVP	11.7	81.1	31.0	10.1	81.3
Female	Heterozygote and homozygote	Overall	22.9	50.3	43.3	3.1	65.6
		UKB	28.8	42.3	51.3	1.4	88.9
		AoU	21.4	56.3	50.0	5.7	33.2
		BioVU	21.9	49.3	31.4	1.5	44.4
		MVP	22.4	51.1	43.1	3.3	77.9
	Homozygote	Overall	1.5	76.2	6.2	13.4	33.9
		UKB	2.4	78.3	7.8	6.5	33.3
		AoU	0.8	66.7	1.5	16.7	5.6
		BioVU	1.8	77.3	4.0	4.5	11.1
		MVP	1.4	76.2	7.5	15.7	54.2

UKB was the cohort where the model was trained. AoU, BioVU, and MVP were the cohorts in which the model was evaluated and validated.

*AoU*, All of Us; *BioVU*, Vanderbilt BioVU; *MVP*, Million Veterans Program; *UKB*, UK Biobank.

To assess utility when applied at population scale, we applied our algorithm to 48,031 people who self-identify as Black race with type 2 diabetes within the Vanderbilt Synthetic Derivative. The model identified substantial subpopulations requiring enhanced screening: 64.2% met possible and 1.1% met likely G6PD-deficient criteria. These groups showed higher 20-year diabetic retinopathy rates (possible: 13.9%; likely: 14.3%) compared with the general population (11.2%). After adjustment, both classifications predicted increased retinopathy risk (likely hazard ratio (HR): 1.41, 95% CI: 1.12-1.78, *P* = .003; possible HR: 1.35, 95% CI: 1.28-1.42, *P* = 2 × 10^−16^). Both diagnoses and treatment occurred later among high-risk subgroups.

## Discussion

Recent research studies have shown those harboring the G6PD p.Val68Met variant face potential diabetes undertreatment due to their lower HbA1c levels relative to blood glucose.^3^^,^^4^ However, translating this finding into clinical care remains challenging because few with G6PD deficiency—we estimate <0.5% via clinical diagnosis codes—are aware of their enzymopathy. This proof-of-concept study demonstrates how readily available clinical data can identify vulnerable populations who may benefit from targeted screening for G6PD deficiency and intervention programs to monitor their diabetes risk and severity using fasting glucose, continuous glucose monitors, or fructosamine. Prior work by Chang et al^10^ demonstrated that the ratio of fasting glucose to hemoglobin A1c is predictive of G6PD deficiency status in a Chinese population, achieving an AUROC of 0.69 in nondiabetic subjects. We tested this approach in our UK Biobank cohort and found similar performance (AUROC 0.65 ± 0.02), but this was significantly inferior to our GG and RDW model (AUROC 0.82 ± 0.03). The GG metric may provide superior discrimination compared with the simple ratio, particularly in populations with the p.Val68Met variant.

Our population health strategy offers distinct advantages for health system implementation, requiring only routine laboratory measurements while providing flexible classification thresholds for different screening scenarios. The “possible G6PD deficiency” criteria prioritize sensitivity over specificity because RDW ≤ 12 fL may capture individuals with normal variation in RDW or other causes of decreased RDW unrelated to G6PD deficiency. This design choice explains the moderate precision (∼31% for male hemizygotes) but high recall (∼81%) of the “possible” criteria, making them suitable for population-level screening, in which capturing most at-risk individuals is prioritized over precision. The particularly low precision of the “possible” criteria for female homozygotes (6.2%) reflects both their low prevalence across cohorts (∼1.5%) and reduced penetrance because of X-chromosome inactivation patterns. On the other hand, the “likely G6PD deficiency” criteria identify populations with 6-fold enrichment of those with the G6PD p.Val68Met variant for focused testing programs. Furthermore, we identified a population of patients with diabetes within the health care system at increased risk for retinopathy. Although we cannot definitively know their G6PD deficiency status, their retinopathy risk may be at least partially attributed to undertreatment, which may be remedied by testing high-risk groups and using plasma glucose for diabetes monitoring, as recommended by the American Diabetes Association.^6^ The absolute risk difference of 3.1% for diabetic retinopathy over 20 years represents a number needed to harm of approximately 32, which may have modest clinical impact at the individual level but may be meaningful at the population scale, given the prevalence of G6PD deficiency.

Our approach is not without limitations. First, our algorithm shows reduced performance for detecting female homozygotes, with precision of only 6.2% using “possible” criteria because X-inactivation in females and low prevalence make laboratory-based screening approaches inherently challenging for this subgroup.^11^ This underscores that our algorithm is most effective for identifying male hemizygotes, who represent most clinically affected individuals. Second, we focus on a single variant in people self-identifying as Black race because clinicians applying this algorithm will be presently unaware of genetic ancestry.

Our algorithm serves as a population-level screening tool to systematically identify populations via electronic health records who may benefit from definitive biochemical or genetic testing. Our approach more generally provides a model for identifying vulnerable populations who may benefit from targeted genetic testing. Future studies should evaluate the effectiveness and implementation outcomes of this population health approach within gLHSs. Identifying, implementing, and critically evaluating such algorithms and interventions across diverse practice settings is a crucial step to advance both genetic medicine and health equity.

## Data Availability

All data are available on the UK Biobank Access Management System https://www.ukbiobank.ac.uk/enable-your-research/apply-for-access, All of Us Researcher Workbench https://www.researchallofus.org/, Vanderbilt BioVU Terra.bio cloud environment, and Million Veterans Program Research Hub https://www.mvp.va.gov/pwa/discover-mvp-data

## Conflict of Interest

Lawrence S. Phillips declares that there is no duality of interest associated with this manuscript. With regard to potential conflicts of interest, Lawrence S. Phillips has served on Scientific Advisory Boards for Janssen and has or had research support from Merck, Pfizer, Eli Lilly, Novo Nordisk, Sanofi, PhaseBio, Roche, Abbvie, Vascular Pharmaceuticals, Janssen, Glaxo SmithKline, and the Cystic Fibrosis Foundation. Lawrence S. Phillips is also a cofounder and Officer and Board member and stockholder for a company, Diasyst, Inc, which markets software aimed to help improve diabetes management. All other authors declare no conflicts of interest.
